# Use of antibacterials in the management of symptoms of acute respiratory tract infections among children under five years in Gulu, northern Uganda: Prevalence and determinants

**DOI:** 10.1371/journal.pone.0235164

**Published:** 2020-06-23

**Authors:** Hindum Lanyero, Jaran Eriksen, Celestino Obua, Cecilia Stålsby Lundborg, Sarah Nanzigu, Agaba Katureebe, Joan N. Kalyango, Moses Ocan

**Affiliations:** 1 Department of Pharmacology and Therapeutics, Makerere University College of Health Sciences, Kampala, Uganda; 2 Department of Public Health Sciences, Karolinska Institutet, Stockholm, Sweden; 3 Department of Laboratory Medicine, Karolinska Institutet, Karolinska University Hospital Huddinge, Stockholm, Sweden; 4 Mbarara University of Science and Technology, Mbarara, Uganda; 5 Infectious Diseases Research Collaboration, Kampala, Uganda; 6 Department of Pharmacy, Makerere University College of Health Sciences, Kampala, Uganda; 7 Clinical Epidemiology Unit, Makerere University College of Health Sciences, Kampala, Uganda; University of Tennessee Knoxville, UNITED STATES

## Abstract

Inappropriate use of antibacterials is a major public health challenge as it can promote emergence of resistance, wastage of financial resources, morbidity and mortality. In this study, we determined the prevalence and factors associated with antibacterial use in managing symptoms of acute respiratory tract infections (ARIs) in households in rural communities of Gulu district, northern Uganda. A cross-sectional study was conducted among households selected using multi-stage sampling. Data were collected through interviews with care-givers of children under five years, using a structured interviewer administered questionnaire. Out of the 856 children who had symptoms of ARIs, 515 (60.2%; CI: 54.5%-65.6%) were treated with antibacterials. The most commonly used antibacterials were amoxicillin (55.2%, n = 358), cotrimoxazole (15.4%, n = 100) and metronidazole (11.4%, n = 74). The determinants of antibacterial use included; getting treatment from a health facility (AOR: 1.85, CI: 1.34–2.56, *P* < 0.001), households located in peri-urban area (AOR: 2.54, CI: 1.34–4.84, *P* = 0.005), and a child having cough (AOR: 7.02, CI: 4.36–11.31, *P* < 0.001). The prevalence of antibacterial use among children under five years with symptoms of ARIs is high in communities of Gulu district, northern Uganda. Getting treatment from a health facility, if a household was located in a peri-urban area and having a cough are positive predictors of antibacterial use. There is need for targeted education on appropriate antibacterial use in rural communities and hospital settings where over prescription is most likely especially in treating symptoms of ARIs among children under five years.

## Introduction

Antibacterials are a valuable resource, the benefit of their use should however be weighed against the potential risk both to the society and the patient [1, 2]. A number of factors need to be considered when deciding to use antibacterials in managing acute respiratory tract infections (ARIs) [3]. The first being the microbiology, as the infection can be bacterial, fungal, viral, parasitic or mixed [4] and the second being the need for antibacterials, since most of these conditions are self-limiting [5].

Acute respiratory tract infections (ARIs) are usually viral, especially in children under five years, and do not require use of antibacterials [6]. However, studies have reported that in most cases these conditions tend to be treated inappropriately with antibacterials [7–9]. A study in Kampala, Uganda reported a 43% prevalence of antibacterial use in treating children under five years with symptoms of ARIs [7]. This high prevalence of antibacterial use is a major public health concern, since it potentially promotes emergence of bacterial resistance, poor clinical outcomes, increased mortality and wastage of financial resources [10].

Factors influencing inappropriate antibacterial use among children under five years include, self-medication, financial limitations, poor access to healthcare services, lack of diagnostic tools, non-adherence to treatment guidelines, workload among healthcare workers, prescribing as per patient expectations and weak regulatory systems [10, 11]. A study carried out to determine factors that influence antibacterial prescribing for ARIs in primary care facilities reported that, healthcare providers are highly influenced to prescribe antibacterials by patient expectations, clinical uncertainty and workload induced time pressures [10]. A study in Kampala, Uganda reported that location of household, level of education of child care-giver, confidence to self-diagnose ARIs, treatment seeking behavior and access to antibacterials; predict self-medication with antibacterials in children under five years with symptoms of ARIs [7].

Northern Uganda suffered more than two decades of armed conflict which affected the healthcare infrastructure. The region has disproportionately low attraction and retention of healthcare professionals in addition to lacking functional healthcare facilities especially in rural communities. While inappropriate use of antibacterials in children under five years has been documented in most low and middle income countries (LMICs), little is known of the prevalence of antibacterial use in children under five years with symptoms of ARIs in northern Uganda [7, 8]. This study therefore investigated prevalence of, and factors associated with, use of antibacterials in management of symptoms of ARIs in children under five years in rural communities of Gulu district, northern Uganda.

## Materials and methods

### Study design, site and population

A cross-sectional household survey was carried out from November 2018 to February 2019 in rural communities of Gulu district, northern Uganda. Gulu is a district in northern Uganda. The national census in 2014 estimated Gulu’s population at 152,276. The economic activity of 90% of the population is subsistence agriculture [12]. For more than two decades of armed conflict the population of Gulu lived in displaced peoples’ camps and protected villages. The district is still recovering (especially the rural areas) from the effects of war which affected the whole society including the health sector. Data were collected from child care-givers in households with children under five years who had displayed symptoms of ARIs within two weeks preceding the data collection date.

### Sample size determination

The minimum sample size required for the study was 865 children under five years. This was computed based on the formula for estimation of a single proportion with adjustment of sample size to cater for multiple stage cluster sampling [13]. The parameters used included: proportion of children getting antibacterial treatment for ARI in rural communities (assumed to be 50% using the conservative approach), 95% level of confidence, sample design effect of 2.0, and 10% adjustment for non-response.

### Sampling criteria

Gulu was purposively selected having been the epicenter of more than two decades of armed conflict in northern Uganda. Within the district, multi-stage sampling was used to select the sub-counties, villages and households with children under five years reported to have had symptoms of ARIs (Appendix 4 in S3 Appendix). Gulu district is administratively divided into Gulu municipality and Aswa county. Gulu municipality has 4 sub-counties (Laroo, Bardege, Layibi and Pece) and Aswa county has 6 sub-counties (Paicho, Awach, Bungatira, Unyama, Patiko and Palaro). In each administrative unit, sub-counties were purposively selected to represent high and low population areas. In Gulu municipality, Laroo (low population size) and Bardege (high population size) were purposively selected. In Aswa county, Awach, Paicho (low population size) and Bungatira, Unyama (high population size) were purposively selected. A total of six (6) sub-counties were purposively selected from Gulu municipality and Aswa county.

In each of the 6 sub-counties, probability proportionate to size (PPS) was applied in estimating the number of villages per sub-county and number of households per village to be sampled [14]. Population size estimates of Gulu district from the 2014 census [12] was used in sample size calculation.

The following formula was used in calculating the number of villages per sub-county and number of households in each village to be sampled [14];

Probability1(Prob1)=a*d/b

Probability2(Prob2)=c/a

Overallweight=1/(Prob1*Prob2)

Where; a = number of individuals in each cluster, b = sum of individuals in all clusters, c = number of individuals sampled per cluster, d = number of sampled clusters, Prob1 = Probability of selection for each sampled cluster and Prob2 = Probability of selection for each individual in each of the sampled clusters.

A total of 44 villages were selected based on PPS criteria, in each village 20 households were selected and in each household, one child who had or had had symptoms of ARI was selected for inclusion into the study. When a household did not have a child under five years who had or had had symptoms of ARIs in the previous two weeks or the care-giver refused to give consent, they were not included in the study. If the household had more than one child under five years that met the inclusion criteria simple random sampling was used to select one child to include in the study.

In each village, we visited all the identified households as per the estimated cluster size with children under five years who met the inclusion criteria before moving to the next village.

### Data collection tool

An interviewer administered questionnaire was used for data collection. The tool was adapted from two different tools after getting permission; a household survey on self-medication with antimicrobials in northern Uganda [15] and a household survey to assess the diffusion of the change of first line antimalarial drug from chloroquine (CQ) to sulphadoxine/pyrimethamine (SP) in a rural district of Tanzania [16]. The tool was pre-tested in the neighboring district of Omoro and modified to capture all variables of interest. Omoro was until 2015 a county in Gulu district and it was equally affected by the over two decades of armed conflict. The population in this neighboring district is structurally and socially similar to that of Gulu district as they are all one tribe (Acholi) found in the greater northern region of Uganda.

### Data collection

Child care-givers for each of the selected children were interviewed using a pre-tested interviewer administered questionnaire. The data collection team was divided into three groups comprising of two people, each group had at least one pharmacy technician (health professional with diploma in pharmacy). The interviews lasted about 30 minutes.

Information on the following variables were collected; sub-county, age of child, age of care-giver, sex of child, sex of care-giver, marital status of care-giver, occupation of care-giver, educational level of care-giver, number of children under five in a household, number of people in household, distance to the nearest health facility and relation of child to the care-giver, symptoms of disease, medication given and the source of the medicine used. We further collected information on the medication they used, and whether the medicines were used as a result of self-medication or prescribed by a health care professional.

### Data management

Questionnaires were reviewed for completeness at the end of each data collection day or shortly after the field work. In case of inconsistencies, research assistants or care-givers were consulted for clarification.

Double data entry was done in Epi-Data 3.1 software. The two datasets were reconciled by comparing them for each field in the questionnaire and in case of any discrepancies, the corresponding questionnaire was checked to establish the correct entry. Data were then imported into STATA 14/IC (Stata Inc., Texas USA) for analysis.

### Statistical analysis

Descriptive statistics were presented using means and standard deviation (SD) for continuous variables. Frequency distributions for selected participant characteristics were done. Crude factor-specific prevalence of antibacterial use was computed using clustered robust standard errors specifying village as the clustering variable. The factors considered were selected participant characteristics/independent variables.

Random effects logistic regression was used for uni-variable analysis to assess simple associations between exposure and outcome variables adjusting for clustering variable, village. In this analysis antibacterial use was considered as the outcome variable and the exposure variables considered were age of child, gender of child, source of treatment, location of household, cough, fast breathing, diarrhea and runny nose. Using the same statistical approach, some factors were explored in a bi-variable analysis for potential confounding by adjusting for each factor and assessing for a change in the odds ratios. Factors that were found to affect the odds ratios by approximately 0.1 were considered potential confounders [17]. This procedure was used to select factors for inclusion and final assessment in multivariable analysis.

All factors which achieved a p-value of less than 0.1 were considered fit to be included in the multivariable model. Gender of a child was included as a *priori* in the model, because some studies have reported gender as a determinant for antibacterial use [18]; although it had no evidence of association with antibacterial use and did not affect the relationship between other factors and antibacterial use in this study. To account for clustering, we used random-effect logistic regression model and likelihood ratio test. The model was built using backward elimination algorithm and statistical significance of the explanatory variables were assessed using likelihood ratio test. Effect modification was assessed by fixing interaction terms between health facility treatment and child age categories, and between cough and child age categories. Appropriateness of the model was tested using the Hosmer and Lemeshow test. All statistical inferential frame works were based on the two-sided P-value and a 5% error margin and analysis was performed using Stata 14.0/IC (Stata Inc., Texas USA).

### Ethics statement

The study was approved by Makerere University School of Biomedical Sciences Research and Ethics Committee (reference SBS-570) and the Uganda National Council of Science and Technology (reference HS235ES). Written informed consent was obtained from care-givers of children under five years prior to data collection (Appendix 2 in S1 Appendix).

## Results

### Socio-demographic characteristics of care-givers and children under five years

A total of 856 households had children who met the inclusion criteria, and care-givers who consented to the study, a response rate of 98.9% (856/865). Over half, 52.1% (n = 446) of the children were male and 96.7% (n = 828) of the care-givers were female. The average age of children under five years was 30±17 months while that of the care-givers was 40±15 years. The care-givers were mostly not educated (57%; n = 488) and peasant farmers (62.1%; n = 532). The households visited were mainly in rural areas (84.6%; n = 724) with majority (71.6%; n = 613) being within 1-hour walking distance to the nearest health facility (Table 1).

**Table 1:** Distribution of antibacterial use by socioeconomic and demographic characteristics of children under five years and care givers in rural communities of Gulu district, northern Uganda (November, 2018–February, 2019).

Variable	Description	Respondents frequency, n (%)	Proportion of antibacterial use, n (%)	95% CI
Age of child (months)	1–12	227 (26.5)	129 (25.0)	20.6–30.1
	13–36	417 (48.7)	239 (46.4)	40.6–52.3
	37–59	212 (24.8)	147 (28.5)	24.5–32.9
Gender of child	Male	446 (52.1)	265 (51.5)	47.4–55.5
	Female	410 (47.9)	250 (48.5)	44.5–52.6
Location of household	Rural	724 (84.6)	415 (80.6)	61.9–91.4
	Peri-urban	132 (15.4)	100 (19.4)	8.6–38.1
Education of care-giver	No education	488 (57.0)	290 (56.3)	48.1–64.2
	Primary level	229 (26.8)	139 (27.0)	22.2–32.4
	O- Level	66 (7.7)	42 (8.2)	5.9–11.2
	A- Level	15 (1.9)	8 (1.6)	0.6–3.9
	Tertiary level	58 (6.8)	36 (7.0)	4.1–11.7
Age of care- giver (years)	15–24	312 (36.5)	173 (33.6)	28.9–38.6
	25–34	336 (39.3)	212 (41.2)	36.8–45.7
	34–44	133 (15.5)	83 (16.1)	13.7–18.8
	45 and above	75 (8.8)	47 (9.1)	6.4–12.8
Gender of care- giver	Female	828 (96.7)	497 (96.5)	94.5–97.8
	Male	28 (3.3)	18 (3.5)	2.2–5.5
Occupation	Peasant	532 (62.1)	310 (60.2)	49.0–70.4
	Skilled Labor	63 (7.4)	41 (8.0)	5.3–11.8
	Business Owner	183 (21.4)	119 (23.1)	17.2–30.4
	Unskilled Labor	78 (9.1)	45 (8.7)	5.8–13.0
Time to reach health facility	Within 1 Hour	613 (71.6)	365 (70.9)	63.3–77.5
	Within 2 Hours	169 (19.7)	105 (20.4)	15.7–26.1
	≥ 3 Hours	74 (8.6)	45 (8.7)	5.6–13.3
Treatment at health facility	Yes	451 (52.7)	295 (57.3)	49.9–64.3
	No	405 (47.3)	220 (42.7)	35.7–50.1

%: Percentage

CI: Confidence Interval

n: Sample size

O-Level: Ordinary Level

A-Level: Advanced Level

### Prevalence of antibacterial use in the treatment of children under five years with symptoms of acute respiratory tract infection

Out of 856 children under five years who had symptoms of ARIs, 515 (60.2%; CI: 54.5–65.6) were treated with antibacterials. Among children, the prevalence of antibacterial use was higher in males (51.5%; CI: 47.4–55.5), those in the age group of 13–36 months (46.4%; CI: 40.6–52.3) and in those who got treatment from a health facility (57.3%; CI: 49.9–64.3). Among child care-givers, the prevalence of antibacterial use was higher if the care-giver was a female (96.5%; CI: 94.5–97.8), had no education (56.3%; CI: 48.1–64.2), peasant (60.2%; CI: 49.0–70.4), and belonged to the age group of 25–34 years (41.2%; CI: 36.8–45.7). The prevalence of antibacterial use was higher in households in rural areas (80.6%; CI: 61.9–91.4) (Table 1).

#### Symptoms of ARIs commonly associated with treatment with antibacterials

**Fig 1:**
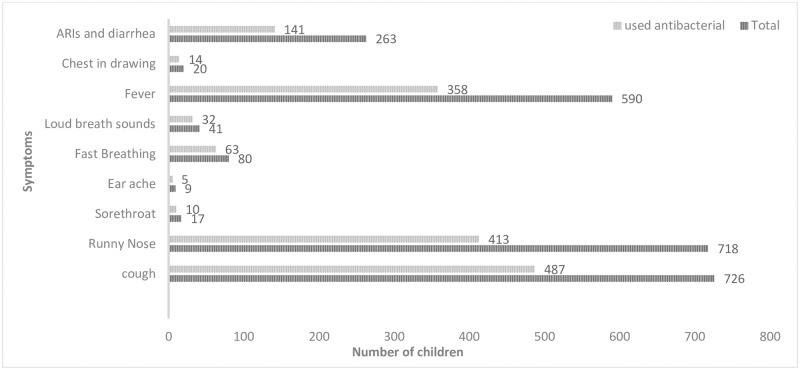
Proportion of children with symptoms of ARIs and antibacterial use.

The symptoms most commonly associated with the use of antibacterials include; runny nose (58%, n = 413), cough (67%, n = 487), fever (61%, n = 358), fast breathing (79%, n = 63) and having symptoms of ARIs with diarrhea (54%, n = 141) (Fig 1).

#### Antibacterials used in treatment of children under five years with symptoms of ARIs

**Fig 2:**
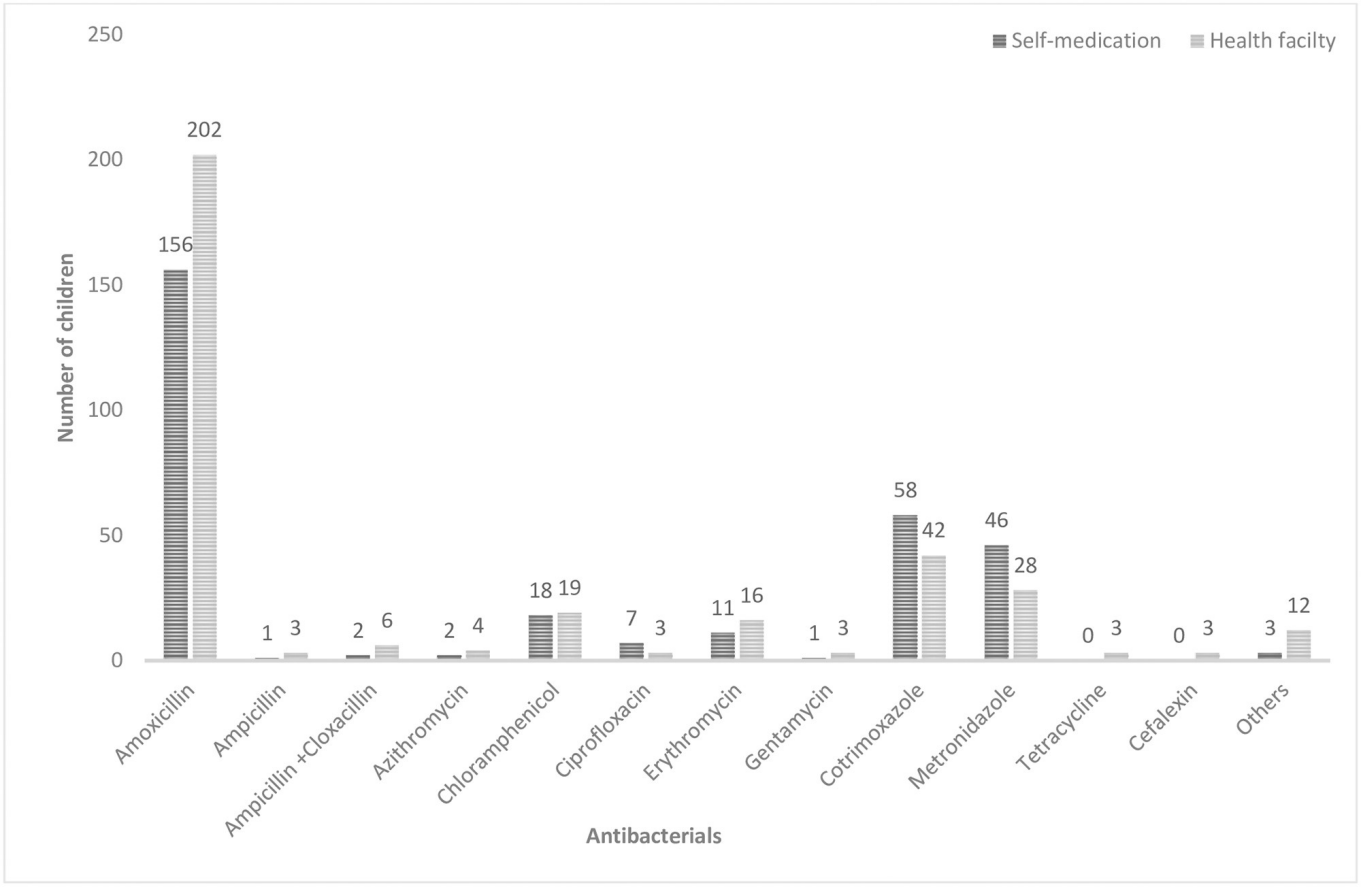
Antibacterials which are commonly used to manage ARIs.

The most commonly used antibacterials to manage ARIs in children under five years were amoxicillin (55.2%, n = 358), cotrimoxazole (15.4%, n = 100) and metronidazole (11.4%, n = 74) (Fig 2).

#### Factors associated with antibacterial use in treatment of children under five years with symptoms of ARIs

In uni-variable analysis, we found that children in the age group 37–59 months (*P* = 0.039), households in peri-urban areas (*P* = 0.017), getting treatment from a health facility (*P* < 0.001), cough (*P* < 0.001), runny nose (*P* = 0.003) and fast breathing (*P* = 0.002) were significantly associated with antibacterial use (Table 2).

**Table 2:** Uni-variable analysis of factors associated with antibacterial use in the treatment of symptoms of ARIs among children under five years in rural communities of Gulu district, northern Uganda (November, 2018–February,2019).

Variable	Category	COR (95% CI)	P-value
Age of child (months)			
	13–36	0.92 (0.65–1.30)	0.637
	37–59	1.55 (1.02–2.35)	0.039
	1–12	Reference group	
Sex of child			
	Female	1.06 (0.80–1.42)	0.682
	Male	Reference group	
Treatment at Health facility			
	Yes	0.56 (0.41–0.75)	<0.001
	No	Reference group	
Location of household			
	Peri-urban	2.20 (1.15–4.21)	0.017
	Rural	Reference group	
Cough			
	Yes	7.98 (4.99–12.76)	<0.001
	No	Reference group	
Fast Breathing			
	Yes	2.58 (1.41–4.72)	0.002
	No	Reference group	
Diarrhea			
	Yes	1.35 (0.99–1.85)	0.060
	No	Reference group	
Runny nose			
	Yes	0.52 (0.34–0.80)	0.003
	No	Reference group	

COR: Crude Odds Ratio

CI: Confidence Interval

%: Percentage

#### Determinants of antibacterial use in treatment of children under five years with symptoms of ARIs

Location of residence and having diarrhea were found to be negative confounders of the association between health facility medication and antibacterial use, while having a cough was a positive confounder of the association between health facility medication and antibacterial use. Fast breathing, runny nose and child age were found to be positive confounders of the association between cough and antibacterial use. We also found that heath facility medication confounds the association between area of residence and antibiotic use (Appendix 5 in S4 Appendix).

Multivariable logistic regression model was used to establish predictors of antibacterial use in children under five years with symptoms of ARIs in rural communities of Gulu district, northern Uganda. Children in peri-urban settings were twice more likely to be given antibacterials compared to those in rural settings (AOR: 2.54, CI: 1.34–4.84). Children who were taken to a health facility (AOR: 1.85, CI: 1.34–2.56) were more likely to use antibacterials compared to those who were not taken to the hospital (AOR: 1.85, CI: 1.34–2.56). Children who had cough were seven times more likely to be given antibacterials compared to those who did not have cough (AOR: 7.02, CI: 4.36–11.31) (Table 3).

**Table 3:** Multi-variable analysis of determinants of antibacterial use in the treatment of symptoms of ARIs among children under five years in rural communities of Gulu district, northern Uganda (November, 2018 –February, 2019).

Variable	Description	AOR (95%CI)	P-value
Age of child(months)	13–36	0.95 (0.65–1.38)	0.781
	37–59	1.21 (0.77–1.91)	0.406
	1–12	Reference group	
Gender of child	Female	1.05 (0.77–1.43)	0.759
	Male	Reference group	
Location of household	Peri-urban	2.54 (1.34–4.84)	0.005
	Rural	Reference group	
Treatment at health facility	Yes	1.85 (1.34–2.56)	<0.001
	No	Reference group	
Cough	Yes	7.02 (4.36–11.31)	<0.001
	No	Reference group	
Fast breathing	Yes	1.87 (0.98–3.55)	0.056
	No	Reference group	
Diarrhea	Yes	0.71 (0.50–1.00)	0.054
	No	Reference group	
Runny nose	Yes	0.73 (0.47–1.14)	0.168
	No	Reference group	

AOR: Adjusted Odds Ratio

CI: Confidence Interval

%: Percentage

## Discussion

Antibacterial use is common (60.2%) in the treatment of children under five years with symptoms of ARIs in communities of Gulu, northern Uganda. Antibacterials are prescription only medicines according to Uganda National drug policy [19]. However, the inadequate enforcement of regulation of access to antibacterials especially in the private sector coupled with non-functional microbiology laboratories in most health facilities could be responsible for the high antibacterial use in children with symptoms of ARIs observed in this study. The high antibacterial resistance selection pressure created by high antibacterial use is a potential driver of resistance emergence and spread (20), further diminishing the already limited choices of antibacterial therapy for infectious diseases especially in LMICs (10). The high prevalence of antibacterial use reported in this study is similar to reports of previous studies done in LMICs [3, 7–9, 20–33].

A household survey in Kampala, Uganda, reported lower prevalence (43%) of antibacterial use in treating children under five years compared to the current study [7]. This could be because the study was done in an urban setting and it only assessed self-medication; whereas our study was done in a rural setting and both self-medication and health care facility use of antibacterials were assessed.

Another study in Vietnam reported a higher prevalence of antibacterial use (71% for mild ARIs and 86% for severe ARIs) compared to the current study [22]; unlike our study where we assessed both self-medication and prescription use of antibacterials in management of children under five years with symptoms of ARIs, the Vietnam study only assessed prescription antibacterial use.

In this study, amoxicillin, co-trimoxazole and metronidazole were the most commonly used antibacterials, a finding similar to that of previous studies in LMICs [7, 9, 29–31]. A survey in Namibia [34] found cotrimoxazole, amoxicillin and azithromycin as the most used antibacterials. A systematic review of antibiotic self-medication in Southeastern Asian region reported amoxicillin, azithromycin, ciprofloxacin, cephalosporins and metronidazole as being the most commonly used antibacterials [32]. The high level of use of these antibacterials in these communities could be due to affordability, availability and ease of accessibility. High antibacterial use is a risk factor for emergence and spread of antibacterial resistance, and there are reports of high resistance to these commonly used antibacterials [35, 36].

We found that antibacterial use is more common in children who are taken to a health facility with symptoms of ARIs. This finding is similar to that from a study in Nigeria which reported a higher prevalence of prescription antibacterial use (48.5%) [20]. In most health facilities especially in LMICs, treatment is based on presumptive diagnosis this coupled with the high prevalence of infectious diseases could be responsible for the high prescription of antibacterials among clinicians in the current study. Furthermore, poor access to the healthcare facilities potentially delay appropriate treatment which worsen symptoms of ARIs thus necessitating use of antibacterials [37–39]. The indiscriminate use of antibacterials in the treatment of ARIs by healthcare workers leads to wastage of resources in an already resource constrained country like Uganda. We also observed that living in less remote (peri-urban) areas was associated with high frequency of antibacterial use compared to rural areas, a finding similar to that of a study in Kampala, Uganda, whereby children in urban areas were more likely to be treated with antibacterials compared to their counter parts in peri-urban areas [7]. This could be because the peri-urban population is more enlightened (7, 17,23) and has better access to health care services, as compared to those in rural areas.

Having symptoms such as cough or fast breathing were associated with antibacterial use in this study, a finding similar to that of previous studies [7, 25–28]. This could be because cough or fast breathing are considered symptoms of pneumonia, and treatment guidelines recommend use of antibacterials [40–42].

The results of our study should be considered in light of some limitations. For example, we used self-reports to establish antibacterial use in children, this is likely to be affected by recall bias. However, a recall period of 14 days has been found to reduce the risk of recall bias [43, 44]. In the current study we used a recall period of 14 days which is likely to have reduced the potential risk of recall bias. In addition, the study could have also been affected by social desirability bias during field data collection. Some of the respondents also did not know the name of the medicines that they had given to their children, this was addressed by requesting the respondents to describe the medicine (color, how many times it was given, taste, whether capsule or tablet) in addition, the respondents were requested to bring the primary or secondary packets of the medicines or the remaining medicine if it was available.

## Conclusions

The prevalence of antibacterial use among children under five years with symptoms of ARIs is high in communities of Gulu district, northern Uganda. Getting treatment from a health facility, if a household was located in a peri-urban area and having a cough are positive predictors of antibacterial use. There is need for targeted education on appropriate antibacterial use in rural communities and hospital settings where over prescription is most likely especially in treating symptoms of ARIs among children under five years.

## Supporting information

S1 Checklist STROBE statement—Checklist of items that should be included in reports of cross-sectional studies. (DOC)

S1 Appendix Informed consent form for sub-study 1. (DOCX)

S2 Appendix Questionnaire for household study on antibacterial use in children under five years 2018. (DOC)

S3 Appendix (DOCX)

S4 Appendix (TXT)
