# Supplementary material for: Use of antibacterials in the management of symptoms of acute respiratory tract infections among children under five years in Gulu, northern Uganda: Prevalence and determinants
Source: PLoS One. 2020 Jun 23;15(6):e0235164. doi: 10.1371/journal.pone.0235164 (PMC7310710; doi:10.1371/journal.pone.0235164)
Supplement: S1 Checklist — (DOC) [file pone.0235164.s001.doc]

STROBE Statement—Checklist of items that should be included in reports of ***cross-sectional studies***

|  | Item No | Recommendation |
| --- | --- | --- |
| **Title and abstract** | 1 | (*a*) Indicate the study’s design with a commonly used term in the title or the abstract  **……………..................................... Page 2, line 28……………………………….....** |
|  |  | (*b*) Provide in the abstract an informative and balanced summary of what was done and what was found  **…………………………………. Page 2, lines 23-41………………………………..** |
| Introduction | | |
| Background/rationale | 2 | Explain the scientific background and rationale for the investigation being reported  **…………………………….. .pages 3-4, lines 47-74………………………………….** |
| Objectives | 3 | State specific objectives, including any prespecified hypotheses  **……………………………... pages 4, lines 74-77………………………………..** |
| Methods | | |
| Study design | 4 | Present key elements of study design early in the paper  **……………………………… page 4, lines 80-88 …………………………………** |
| Setting | 5 | Describe the setting, locations, and relevant dates, including periods of recruitment, exposure, follow-up, and data collection  **……………… page 4, lines 80-81… pages 7-8, lines 140-157……………………** |
| Participants | 6 | (*a*) Give the eligibility criteria, and the sources and methods of selection of participants  **………………………….. page 6, lines 121-128………………………………….** |
| Variables | 7 | Clearly define all outcomes, exposures, predictors, potential confounders, and effect modifiers. Give diagnostic criteria, if applicable  **…..pages 7-8, lines 144-157…page 9, lines 187-189..pages 13-14, lines 251-256.….** |
| Data sources/ measurement | 8* | For each variable of interest, give sources of data and details of methods of assessment (measurement). Describe comparability of assessment methods if there is more than one group  **………………… page 7, lines 140-141………………………………………** |
| Bias | 9 | Describe any efforts to address potential sources of bias  **………………………. Page 17, lines 313-324……………………………….** |
| Study size | 10 | Explain how the study size was arrived at  **……………… page 5, lines 90-95…….pages 5-6, lines 108-123……………..** |
| Quantitative variables | 11 | Explain how quantitative variables were handled in the analyses. If applicable, describe which groupings were chosen and why  **…………………… page 8, lines 167-171…………………………….** |
| Statistical methods | 12 | (*a*) Describe all statistical methods, including those used to control for confounding  **………………..pages 8-9, lines 172-187………………………………..** |
|  |  | (*b*) Describe any methods used to examine subgroups and interactions  **…………………page 9, lines 187-189………………………………….** |
|  |  | (*c*) Explain how missing data were addressed  **…………………..page 8, lines 159-161………………………………..** |
|  |  | (*d*) If applicable, describe analytical methods taking account of sampling strategy  **………………….page 8 lines 170-173……………………………………** |
|  |  | (*e*) Describe any sensitivity analyses  **…………………………Not applicable………………………………………..** |
| Results | | |
| Participants | 13* | (a) Report numbers of individuals at each stage of study—eg numbers potentially eligible, examined for eligibility, confirmed eligible, included in the study, completing follow-up, and analysed  **………………..page 10, lines 202-203…………………………** |
|  |  | (b) Give reasons for non-participation at each stage  **………….page 6, line 124…**(Appendix 4 in S4 Appendix 4). **……………** |
|  |  | (c) Consider use of a flow diagram  **………** (Appendix 4 in S4 Appendix 4). **……….** |
| Descriptive data | 14* | (a) Give characteristics of study participants (eg demographic, clinical, social) and information on exposures and potential confounders  **…………………page 10-11, lines 202-219, page 13-14, lines 251-256……………** |
|  |  | (b) Indicate number of participants with missing data for each variable of interest  **………………………………………………………………………………..** |
| Outcome data | 15* | Report numbers of outcome events or summary measures  **………………………page10, lines 212-213………………………………..** |
| Main results | 16 | (*a*) Give unadjusted estimates and, if applicable, confounder-adjusted estimates and their precision (eg, 95% confidence interval). Make clear which confounders were adjusted for and why they were included  **………………………pages 13-14, lines 247-256………………………….** |
|  |  | (*b*) Report category boundaries when continuous variables were categorized  **……………………………………………………………………………** |
|  |  | (*c*) If relevant, consider translating estimates of relative risk into absolute risk for a meaningful time period  **………………………….we used odds ratios…………………………….** |
| Other analyses | 17 | Report other analyses done—eg analyses of subgroups and interactions, and sensitivity analyses  **…………………………… None …………………………………….** |
| Discussion | | |
| Key results | 18 | Summarise key results with reference to study objectives  **……..pages 15-17, lines 266,293,301-303,309………………** |
| Limitations | 19 | Discuss limitations of the study, taking into account sources of potential bias or imprecision. Discuss both direction and magnitude of any potential bias  **……...............page17, lines 313-324………………………….** |
| Interpretation | 20 | Give a cautious overall interpretation of results considering objectives, limitations, multiplicity of analyses, results from similar studies, and other relevant evidence  **……………..pages 15-17, lines 266-324……………………..** |
| Generalisability | 21 | Discuss the generalisability (external validity) of the study results  **……………………page 17, lines 326-331………………………..** |
| Other information | | |
| Funding | 22 | Give the source of funding and the role of the funders for the present study and, if applicable, for the original study on which the present article is based  **……should not appear in the manuscript as per the guidelines of PLoS ONE……** |

*Give information separately for exposed and unexposed groups.

**Note:** An Explanation and Elaboration article discusses each checklist item and gives methodological background and published examples of transparent reporting. The STROBE checklist is best used in conjunction with this article (freely available on the Web sites of PLoS Medicine at http://www.plosmedicine.org/, Annals of Internal Medicine at http://www.annals.org/, and Epidemiology at http://www.epidem.com/). Information on the STROBE Initiative is available at www.strobe-statement.org.
