# Supplementary material for: Use of antibacterials in the management of symptoms of acute respiratory tract infections among children under five years in Gulu, northern Uganda: Prevalence and determinants
Source: PLoS One. 2020 Jun 23;15(6):e0235164. doi: 10.1371/journal.pone.0235164 (PMC7310710; doi:10.1371/journal.pone.0235164)
Supplement: S1 Appendix — (DOCX) [file pone.0235164.s002.docx]

**Appendix 2: INFORMED CONSENT FORM FOR SUB-STUDY 1**

**Title of the proposed study:** MONITORING ANTIBACTERIAL USE IN CHILDREN UNDER FIVE IN RURAL COMMUNITIES OF NORTHERN UGANDA.

**Investigators:**

1. Dr. Hindum Lanyero (Principle investigator) Makerere University College of Health Sciences.
2. Dr. Sarah Nanzigu (Supervisor) Makerere University College of Health Sciences.
3. Dr. Moses Ocan (Supervisor) Makerere University College of Health Sciences.
4. Dr. Jaran Eriksen (Supervisor) Karolinska Institutet.

**Introduction:**

You are being asked to be in a study of ***monitoring antibacterial use in children under five in rural communities of northern Uganda.*** This is a research study and does not involve provision of health care. We are carrying out this study because it has been reported that antibiotics (antibacterial medicines) are being over used and misused when treating children under the age of five years. The problem with not using these medicines in the right way could lead to them not working in future and this is not good because many people will end up dying or will need to use medicines that are expensive so as to get better.

You were selected as a possible participant because you have a child under five years who has had flu, cough and/or diarrhea in the past two weeks. You are not the only one being asked to participate in this study, we are going to interview about 800 people who have children under five years who have had flu, cough and/or diarrhea in the past two weeks.

We ask that you to read or allow to be read to this form and ask any questions that you may have before agreeing to be in the study.

**Purpose:**

The purpose of the study is to find out how antibiotics are used in the community to treat children under five who develop symptoms of flu, cough and/or diarrhea.

This research will be published as a book and presented as papers on antibacterial use in children under five in rural communities which will lead to an award of a PhD degree.

**Description of the Study Procedures:**

If you agree to be in this study we will meet you once and you will be asked to answer questions on the kind of treatment your child received when they had symptoms of flu, cough and/or diarrhea. You will be asked where you got the medication, whether you normally store medicines at home and for what purpose. Your knowledge regarding the management of flu, cough and/or diarrhea will be assessed. In case we later (could be today or after today) discover that there are questions that need to be clarified from your responses we will call you on the number that you have provided or even come back to your home.

**Who will participate in the study?**

Care givers of children under five years and their children under five years who have had symptoms of flu, cough and or diarrhea will participate in the study. The care givers will give information about medicines taken by their children when they had these symptoms.

**Risks/Discomfort:**

This is a minimal risk study with no anticipated problem since it will only involve answering questions.

**Benefits:**

The findings of this study will guide policy makers in coming up with evidence based solutions that will lead to proper use of these medicines. Some of the solutions could involve coming and educating you and other members of the community about appropriate use of medicines hence improving health service delivery to individuals.

**Confidentiality:**

Your involvement is appreciated and confidentiality will be highly observed, your identification will not be required to appear anywhere in the study. Only the principle investigator and co-investigators will access to your records which will be kept in locked cabin. However, overseeing bodies such as the ethics committee can also see this information on request. We will not include any information in any report we may publish that would make it possible to identify you.

**Right to Refuse or Withdraw:**

Your participation is voluntary and you have a right to refuse answering any question that you feel uncomfortable with, you are also free to withdraw from the study at any time without fear of any consequences.

**Payments**

You will not be given any money for participating in this study.

**Questions:**

In case you have any questions that you want to ask or any additional information you want to give regarding this study after today you can reach the principle investigator, Dr. Hindum Lanyero on **0775866653.**

**Questions about participants’ rights:**

In case you have any questions that you want to ask about your rights, you can call the chair, School of Biomedical Sciences Higher Degrees Research and Ethics Committee, Dr. Erisa Mwaka on **0752575050.**

**Declaration of Consent:**

Thank you for reading the information in the consent form and for asking any questions that you might have had. If you would like to participate in the research, please respond to each of the following questions by ticking in the boxes and then signing the form.

Participant study number……………………………… Date……………………………..

Gender………………………………………………………Age…………………………….

**Please tick any box that you agree with**

1. I confirm that I have read and understood the information in the consent form and have had the opportunity to consider the information, ask questions and have had these answered satisfactorily.
2. I understand that my consent is voluntary and that I am free to withdraw at any time without giving any reason without my legal rights being affected.
3. I hereby agree that the Dr. Hindum Lanyero of Makerere University and her research team have the right to use the personal data as indicated in the consent form.

Name of consenting staff Signature Date

………………………………… ………………………………. ……….........................

Name of child’s care giver Signature Date

………………………………… ………………………………. ……….........................

Name of witness Signature Date

**(If care giver can’t read or write)**
