# Supplementary material for: Use of antibacterials in the management of symptoms of acute respiratory tract infections among children under five years in Gulu, northern Uganda: Prevalence and determinants
Source: PLoS One. 2020 Jun 23;15(6):e0235164. doi: 10.1371/journal.pone.0235164 (PMC7310710; doi:10.1371/journal.pone.0235164)
Supplement: S2 Appendix — (DOC) [file pone.0235164.s003.doc]

| **QUESTIONNAIRE FOR HOUSEHOLD STUDY ON ANTIBACTERIAL USE IN CHILDREN UNDER FIVE YEARS 2018** | | | | | | | | | | | | | | | | | | | | | | | | | | | | | | | | | | | | | | | |  | | | | |
| --- | --- | --- | --- | --- | --- | --- | --- | --- | --- | --- | --- | --- | --- | --- | --- | --- | --- | --- | --- | --- | --- | --- | --- | --- | --- | --- | --- | --- | --- | --- | --- | --- | --- | --- | --- | --- | --- | --- | --- | --- | --- | --- | --- | --- |
| **DEMOGRAPHIC CHARACTERISTICS** | | | | | | | | | | | | | | | | | | | | | | | | | | | | | | | | | | | | | | | |  | | | | |
| Questionnaire number | | | | | | | | | | | | | | | | | | | | | | | |  | | | | | | | | | | | | | | | |  | | | | |
| Name of interviewer | | | | | | | | | | | | | | | | | | | | | | | |  | | | | | | | | | | | | | | | |  | | | | |
| Date | | | | | | | | | | | | | | | | | | | | | | | |  | | | | | | | | | | | | | | | |  | | | | |
| Village name | | | | | | | | | | | | | | | | | | | | | | | |  | | | | | | | | | | | | | | | |  | | | | |
| Sub-County name | | | | | | | | | | | | | | | | | | | | | | | |  | | | | | | | | | | | | | | | |  | | | | |
| Tel contact of interviewee | | | | | | | | | | | | | | | | | | | | | | | |  | | | | | | | | | | | | | | | |  | | | | |
| - 1. Age in years of interviewee (In completed years) | | | | | | | | | | | | | | | | | | | | | | | |  | | | | | | | | | | | | | | | |  | | | | |
| - 1. Age of child( In Completed months ≤59) | | | | | | | | | | | | | | | | | | | | | | | |  | | | | | | | | | | | | | | | |  | | | | |
| - 1. Sex of caregiver: | | | | | | | | | | | | | | | | | | | | | | | | (a) Male  | | | | | | | | | | (b) Female  | | | | | |  | | | | |
| - 1. Sex of child: | | | | | | | | | | | | | | | | | | | | | | | | (a) Male  | | | | | | | | | | (b) Female  | | | | | |  | | | | |
| - 1. Marital Status: | | | | | | (a)Never married   | | | | | | | | | | | (b)married /co-habiting  | | | | | | | | | | (c)widowed/er  | | | | | | | (d)divorced/separated | | | | | |  | | | | |
| - 1. Educational status: | | | (a). No formal  education  | | | | | | | | (b) Completed primary school  | | | | | | | | (c). Completed O-level secondary school  | | | | | | | | | | (d). Completed A-level secondary school  | | | | | | | | (e). Completed tertiary education  | | |  | | | | |
| - 1. Occupation | | | | | 1. Peasant    | | | | | | | | | (b). Civil servant | | | | | | | (c). health worker | | | | | | | | (d).Businessman/woman  | | | | | | | | (e)Other  Specify  | | |  | | | | |
| - 1. Number of people living in the household | | | | | | | | | | | | | | ……………………………………………………….. | | | | | | | | | | | | | | | | | | | | | | | | | |  | | | | |
| - 1. Do you have under-five children in your household? | | | | | | | | | | | | | | (a) Yes  if yes how many…………… | | | | | | | | | | | | | | | | | | | (b) No  | | | | | | |  | | | | |
| - 1. Has any child below five in the household been ill in the past two weeks with diarrhea or acute respiratory infection? | | | | | | | | | | | | | | (a) Yes  if yes how many……………  ***(if more than 1 child randomly choose 1)*** | | | | | | | | | | | | | | | | | | | (b) No  | | | | | | |  | | | | |
| **ILLNESS EXPERIENCE** | | | | | | | | | | | | | | | | | | | | | | | | | | | | | | | | | | | | | | | |  | | | | |
| - 1. Who cared for the child during the illness? | | | | | | | | | | | | | | | | | | | | | | | | | | | | | | | | | | | | | | | |  | | | | |
| (a)sister/ brother  | | (b)mother | | | | | | (c)father | | | | | | | (d)Relative | | | | | | | | (e)Friend | | | | | | (f) neighbor | | | | | | | (g)other  (specify) | | | |  | | | | |
| - 1. Which symptoms did the child have during this illness? ***(tick all that apply)***  | 1 | Cough |  | | --- | --- | --- | | 2 | Runny nose |  | | 3 | Sore throat |  | | 4 | Ear ache/ear discharge |  | | 5 | fast breathing |  | | 6 | Inabity to feed |  | | 7 | High pitched breath sound |  | | 8 | Itchy watery eyes |  | | 9 | headache |  | | 10 | fever |  | | 11 | fatigue |  | | 12 | Skin rash |  | | 13 | vomiting |  | | 14 | Sneezing |  | | 15 | Chest in drawing |  | | 16 | Loose stool/ running stomach with no blood |  | | 17 | Loose stool / running stomach with blood. |  | | 18 | other(specify) |  |   **SELF-MEDICATION** | | | | | | | | | | | | | | | | | | | | | | | | | | | | | | | | | | | | | | | |  | | | | |
| **HOUSEHOLD FACTORS**   - 1. a) Did you do anything to manage the child at home without prescription from a health care worker (VHT is also a health worker)?   a) yes  b) No  If No skip to 20 | | | | | | | | | | | | | | | | | | | | | | | | | | | | | | | | | | | | | | | |  | | | | |
| 1. b) if yes what did you do ***(tick all that apply)*** | | | | (a) sponging  | | | | | | (b) gave medicine  | | | | | | | | (c) gave herbal treatment  | | | | | | | | | | | (d) changed eating habits  | | | | | | | (e) other   please specify: | | | |  | | | | |
| 1. a) If you gave drugs in 13b) Which drugs did you give at home? Fill in table below | | | | | | | | | | | | | | | | | | | | | | | | | | | | | | | | | | | | | | | |  | | | | |
| | No. | Name of drug/ medicine or its description (colour, taste, disease for which medicine was used) | How medicine was identified  1.As observed  2.As reported | Formulation of medicines taken  1 tablets 2 capsule  3 vials/injections  4 cream/ointment  5 Eye/ear ointment  6 syrup 7 other (specify) | Number/Amount obtained or stock that was at home | Regimen/ number taken per day | For how many days have you been taking or did child take this medicine | Did any medicines remain after treatment | | | --- | --- | --- | --- | --- | --- | --- | --- | --- | | **Yes** | **No** | | 1 |  |  |  |  |  |  |  |  | | 2 |  |  |  |  |  |  |  |  | | 3 |  |  |  |  |  |  |  |  | | 4 |  |  |  |  |  |  |  |  | | 5 |  |  |  |  |  |  |  |  | | 6 |  |  |  |  |  |  |  |  |  - 1. b) Was the child given an antibiotic agent during the management of illness at home? a) yes  b) No    **This can be filled later by data collector.** | | | | | | | | | | | | | | | | | | | | | | | | | | | | | | | | | | | | | | | |  | | | | |
| 1. c) Where did you get these drugs that you gave at home and who recommended it? ***(tick all that apply)*** | | | | | | | | | | | | | | | | | | | | | | | | | | | | | | | | | | | | | | | |  | | | | |
| |  | **Name of medicine** | **Recommended/initiated by?**  1=Self(care taker)  2=Household member  3=Friend/ neighbor  4=Drug seller/pharmacist  5=Traditional healer  99=others(specify) | **Obtained from**  1=Available at home  2=Friend/neighbor  3=Drug shop/pharmacy  4=Retail shop  5=Traditional healer  99=others(specify) | **How much did you spend?**  (amount of money used to obtain the drugs) | | --- | --- | --- | --- | --- | | 1 |  |  |  |  | | 2 |  |  |  |  | | 3 |  |  |  |  | | 4 |  |  |  |  | | 5 |  |  |  |  | | 6 |  |  |  |  | | | | | | | | | | | | | | | | | | | | | | | | | | | | | | | | | | | | | | | | |  | | | | |
| 14.d) If the child did not complete all the medicine meant for the condition, what was the reason?   | **Reasons for not taking all the medicines meant for the treatment** | | **TICK** | | --- | --- | --- | | 1 | Symptoms improved before medicine was finished |  | | 2 | Lost/misplaced them |  | | 3 | Gave them to another sick member at home |  | | 4 | I needed to keep some at home for future use in case of sickness |  | | 5 | Experienced bad effects/side effects |  | | 6 | I forgot to continue taking the medicines |  | | 7 | The drug was not working/illness continued |  | | 8 | Someone from the household decided the medicines are not needed |  | | 9 | Ongoing treatment. |  | | 14.e) What did you do with the medicine that remained after you stopped taking them? (Tick only one that applies)   | **Handling of left over medicines** | | **Tick** | | --- | --- | --- | | 1 | Threw them away |  | | 2 | Gave them to the neighbour |  | | 3 | Gave them to another sick member of the home |  | | 4 | Kept them for future use |  | | 5 | Returned them where I got them |  | | 6 | Others(specify) |  | | | | | | | | | | | | | | | | | | | | | | | | | | | | | | | | | | | | | | | | | | | |  | | | | |
| - 1. a) For the medicines that you gave your child at home, do you know the following about it?  | **Property/feature of the medicines** | | Tick all that apply | | | --- | --- | --- | --- | | **Yes** | **No** | | 1 | Dose to be taken |  |  | | 2 | Duration of treatment(therapy) |  |  | | 3 | Side effects of the medicines |  |  | | 4 | Interactions of the medicines |  |  | | 5 | Contraindications of the medicine |  |  | | | | | | | | | | | | | | | | | | | | | | | | | | | | | | | | | | | | | | | | |  | | | | |
| 15.b) How did you get information about the drugs?   | **Source of information on the medicines** | | **Tick** | | --- | --- | --- | | 1 | Reading the drug leaflet |  | | 2 | Previous prescriptions |  | | 3 | Drug promotion information(pharmaceutical companies) |  | | 4 | Neighbor/friend/relative |  | | 5 | Experience from previous use of a similar medicine |  | | 6 | Newspaper/TV/Radio |  | | 7 | Others(specify) |  | | | | | | | | | | | | | | | | | | | | | | | | | | | | | | | | | | | | | | | | |  | | | | |
| **HEALTH FACILITY FACTORS**   - 1. How much time (minutes/hours) does it take to walk to the nearest health facility that you usually get treatment? ………………………………………. | | | | | | | | | | | | | | | | | | | | | | | | | | | | | | | | | | | | | | | |  |  |  | | |
| - 1. Designation of the nearest health facility:***(tick nearest apply)*** | | | | | | | (a). Public  | | | | | | | | | (b). Private for profit   (clinic, drug shop, pharmacy) | | | | | | | | | | | | (c)Private not for profit   (PNFP) | | | (d) VHT  | | | | | | | | (e)other   (specify) |  | | |
| - 1. Have you ever treated yourself or any other member of your family from home without consulting a health care provider because of any of the following reasons?      | **Reason** | | **yes** | **No** | | --- | --- | --- | --- | | 1 | Long waiting time at the health center/hospital |  |  | | 2 | Previous success experience using the same drug |  |  | | 3 | Doctor or pharmacist not available |  |  | | 4 | Expensive consultation fees |  |  | | 5 | Lack of health facility |  |  | | 6 | Expensive laboratory fees |  |  | | 7 | Crowding at the health center/hospital |  |  | | 8 | Long distance to the health center/hospital |  |  | | 9 | No drugs at the health center |  |  | | 10 | Other(specify) |  |  | | | | | | | | | | | | | | | | | | | | | | | | | | | | | | | | | | | | | | | | |  | (b). Private for profit   (clinic, drug shop, pharmacy) | (c)Private not for profit   (PNFP) | (d) VHT  | (e)other   (specify) |
| - 1. (a) Did your child heal/get better? | | | | | | | | | | | | | | | | | | | | | | | | | (a) Yes  | | | | | (b) No  | | | | | | | | | |  | | | | |
| 1. (b) If no did you seek care outside the home for this illness****?***   ***(where you took child to get medicine/treatment)*** | | | | | | | | | | | | | | | | | | | | | | | | | (a) Yes  | | | | | (b) No  | | | | | | | | | |  | | | | |
| 1. (c) If yes: Where ***(tick all that apply)*** | | | | | | | | | | | | | | | | | | | | | | | | | | | | | | | | | | | | | | | |  | | | | |
| (a). Drug outlet  /Pharmacy/Drug stores | (b)Private for profit/ clinic  | | | | | | | | | | | (c). Private not for profit  | | | | | | | | (d). Public facility | | | | | | (e). Traditional  healer | | | | | | (f) VHT  | | | | | | (g) Others  specify | |  | | | | |
| **THOSE WHO TOOK CHILDREN TO THE HEALTH FACILITY *( only those who have ticked one of these options above a,b,c,d,f)*** | | | | | | | | | | | | | | | | | | | | | | | | | | | | | | | | | | | | | | | |  | | | | |
| 20. (a) Was medicine given to child where you took them? | | | | | | | | | (a) Yes   b) No  | | | | | | | | | | | | | If yes answer 20.b)  If no stop here. | | | | | | | | | |  | | | | | | | |  | | | | |
| 20.(b) Did they explain to you how medicine should be taken? | | | | | | | | | (a) Healthcare provider gave medicine them self  ***If a is answer go to 23*** | | | | | | | | | | | | | (b) Healthcare provider gave medicine and explained how to give the drugs   ***If b is answer go to 21.c)*** | | | | | | | | | | (c) Healthcare provider gave medicine but did not explain how to give medicine   ***If c is answer go to 21.d)*** | | | | | | | |  | | | | |
| 21. c) If healthcare provider gave medicine and explained how to give the drugs, explain how?   | No. | Name of  medicine | How medicine  was identified  1.As observed  2.As reported | Formulation  1. tablets 2. capsule  3. vials/injections  4. cream/ointment  5. Eye/ear ointment  6. syrup 7. Other | Regimen  ( frequency of taking medicines each day) | Duration  (in days) | Any other instructions  ( e.g after meals, before meals, with milk and any side effects) | | --- | --- | --- | --- | --- | --- | --- | | 1 |  |  |  |  |  |  | | 2 |  |  |  |  |  |  | | 3 |  |  |  |  |  |  | | 4 |  |  |  |  |  |  | | 5 |  |  |  |  |  |  | | | | | | | | | | | | | | | | | | | | | | | | | | | | | | | | | | | | | | | | |  | | | | |
| 21. d) How did you give the child?   | No. | Name of medicine | Formulation  1 tablets 2 capsule  3 vials/injections  4 cream/ointment  5 Eye/ear ointment  6 syrup 7 Other | Regimen  ( frequency of taking medicines each day) | Duration  (In days) | Any other thing you did as you gave child medicine | | --- | --- | --- | --- | --- | --- | | 1 |  |  |  |  |  | | 2 |  |  |  |  |  | | 3 |  |  |  |  |  | | 4 |  |  |  |  |  | | 5 |  |  |  |  |  |   21. e) Please confirm using prescription. Record actual data from prescription: Write N/A if prescription not available   | No. | Name of medicine | Formulation  1 tablets 2 capsule  3 vials/injections  4 cream/ointment  5 Eye/ear ointment  6 syrup 7 Other | Regimen  ( frequency of taking medicines each day) | Duration  (In days) | Any other instruction | Diagnosis(DX) | | --- | --- | --- | --- | --- | --- | --- | | 1 |  |  |  |  |  |  | | 2 |  |  |  |  |  |  | | 3 |  |  |  |  |  |  | | 4 |  |  |  |  |  |  | | 5 |  |  |  |  |  |  | | | | | | | | | | | | | | | | | | | | | | | | | | | | | | | | | | | | | | | | |  | | | | |
|  | | | | | | | | | | | | | | | | | | | | | | | | | | | | | | | | | | | | | | | |  | | | | |
| 21 .f) If the drugs were not given according to the prescriber’s instructions: Why not? ***(tick all that apply)*** | | | | | | | | | | | | | |  | **Reasons for not following the prescribers instructions** |  | | --- | --- | --- | | 1 | Symptoms improved before drug was finished |  | | 2 | Lost/misplaced them |  | | 3 | Gave it to another member of family who was sick |  | | 4 | I kept some for future use in case of sickness |  | | 5 | Experienced bad effects/side effects |  | | 6 | I forgot to give the child medicine |  | | 7 | The drug was not working |  | | 8 | Someone in the household decided medicines not needed |  | | 9 | I did not understand what the health worker told me |  | | 10 | I could not afford all the medicine |  | | 11 | Other(specify) |  | | | | | | | | | | | | | | | | | | | | | | | | | | | |  | | | | |
| 1. g) Was the child given an antibacterial agent during the management illness outside home? | | | | | | | | | | | | | (a) yes  | | | | | | | | | | | | | | | | | | | | | | (b) No  | | | | |  | | | | |
| 1. a) Did any drug remain from the treatment? | | | | | | | | | | | | | (a) yes  | | | | | | | | | | | | | | | | | | | | | | (b) No   If no skip to 23 | | | | |  | | | | |
| 22 b) If yes may I please look at them to me? Write down number of tablets/amount of syrup/injections, name, and how long they have had the medicine: Put N/A if medicine is not available   | No | Name of medicine | Formulation:1 Tablet 2.capsule  3. vials/injections 4.cream/ointment  5.Eye/ear ointment 6.syrup 7.other (specify) | Number of units | How long have you had this medicine in the house ( in days) | | --- | --- | --- | --- | --- | | 1 |  |  |  |  | | 2 |  |  |  |  | | 3 |  |  |  |  | | 4 |  |  |  |  | | 5 |  |  |  |  |   22.c) Why didn’t the child take all the medicines ***(tick all that apply)***   |  | **Reasons for not taking all the medicines obtained** |  | | --- | --- | --- | | 1 | Symptoms improved before drug was finished |  | | 2 | Lost/misplaced them |  | | 3 | Gave it to another member of family who was sick |  | | 4 | I kept some for future use incase of sickness |  | | 5 | Experienced bad effects/side effects |  | | 6 | I forgot to give the child medicine |  | | 7 | The drug was not working |  | | 8 | Someone in the household decided medicines not needed |  | | 9 | Ongoing treatment |  | | 10 | Other(specify) |  | | | | | | | | | | | | | | | | | | | | | | | | | | | | | | | | | | | | | | | | |  | | | | |
