# Supplementary material for: Use of antibacterials in the management of symptoms of acute respiratory tract infections among children under five years in Gulu, northern Uganda: Prevalence and determinants
Source: PLoS One. 2020 Jun 23;15(6):e0235164. doi: 10.1371/journal.pone.0235164 (PMC7310710; doi:10.1371/journal.pone.0235164)
Supplement: S3 Appendix — (DOCX) [file pone.0235164.s004.docx]

| GULU DISTRICT was purposively selected |
| --- |

| 10 Sub-counties in the district |
| --- |

| 6 Sub-counties purposively selected |
| --- |

| 123 Villages in the 6 sub-counties |
| --- |

| 44 Villages randomly selected |
| --- |

| All households with children under five years |
| --- |

| 856 were recruited, 9 care-givers declined to join the study |
| --- |

**Multi-stage sampling**
